# Synthesis and Biological Characterization of a New Norbormide Derived Bodipy FL-Conjugated Fluorescent Probe for Cell Imaging

**DOI:** 10.3389/fphar.2018.01055

**Published:** 2018-09-25

**Authors:** Claudio D'Amore, Genny Orso, Alessia Forgiarini, Giulio Ceolotto, David Rennison, Giovanni Ribaudo, Morgan Jay-Smith, Brian Hopkins, Margaret A. Brimble, Sergio Bova

**Affiliations:** ^1^Department of Pharmaceutical and Pharmacological Sciences, University of Padova, Padova, Italy; ^2^Department of Medicine, University of Padova, Padova, Italy; ^3^School of Chemical Sciences, University of Auckland, Auckland, New Zealand; ^4^Landcare Research, Lincoln, New Zealand

**Keywords:** fluorescent probes, live cell imaging, norbormide, BODIPY, vascular smooth muscle cells, LX2 cells

## Abstract

**Background:** Norbormide (NRB) is a selective rat toxicant endowed with vasoconstrictor activity confined to the rat peripheral arteries. In a recent work we used a fluorescent derivative of NRB (NRB-AF12), obtained by coupling the NBD fluorophore to the parent molecule via a linker, in order to gain information about the possible site of action of the unlabeled compound. We found that NRB-AF12 labeled intracellular organelles in both NRB-sensitive and -insensitive cells and we accordingly proposed its use as a scaffold for the development of a new class of fluorescent probes. In this study, we examined the fluorescent properties of a BODIPY FL-conjugated NRB probe (MC009) developed: (A) to verify if NRB distribution could be influenced by the attached fluorophore; (B) to improve the fluorescent performance of NRB-AF12.

**Methods:** MC009 characteristics were investigated by confocal fluorescence microscopy, in freshly isolated rat caudal artery myocytes (FIRCAM) and in LX2 cells, representative of NRB-sensitive and insensitive cells, respectively.

**Main results:** In both FIRCAM and LX2 cells MC009 stained endoplasmic reticulum, mitochondria, Golgi apparatus and lipid droplets, revealing the same intracellular distribution as NRB-AF12, and, at the same time, had both improved photostability and gave a more intense fluorescent signal at lower concentrations than was possible with NRB-AF12, which resulted in a better and finer visualization of intracellular structures. Furthermore, MC009 was effective in cellular labeling in both living and fixed cells. At the concentration used to stain the cells, MC009 did not show any cytotoxic effect and did not affect the regular progression of cell cycle and division.

**Conclusions:** This study demonstrates that the distribution of fluorescently labeled NRB is not affected by the type of fluorophore attached to the parent compound, supporting the idea that the localization of the fluorescent derivatives may reasonably reflect that of the parent compound. In addition, we observed a marked improvement in the fluorescent properties of BODIPY FL-conjugated NRB (MC009) over its NBD-derived counterpart (NRB-AF12), confirming NRB as a scaffold for the development of new, high performance, non-toxic fluorescent probes for the labeling of intracellular structures in both living and fixed cells.

## Introduction

Norbormide (NRB) is a very unique compound characterized by a species (rat)-, tissue (vascular)-, and stereo (endo isomers)- specific biological activity that is lethal to rats, in which it induces a profound and irreversible vasoconstriction that has been associated to its toxic effect (Roszkowski, 1965; Poos et al., 1966; Bova et al., 2001; Cavalli et al., 2004; Rennison et al., 2007). Previous studies indicated that NRB elicits its vasoconstrictor action by directly interacting with rat vascular myocytes, with no apparent interaction with the vascular endothelium (Fusi et al., 2002). The molecular site of action and the mechanisms involved in the vasoconstrictor effect of NRB are unknown; however, it can be hypothesized, based on the features of its biological effect, the existence of a target that is exclusively expressed in the rat artery myocytes, or one that is part of a family expressed in the blood vessels of all animal species, but which has species-selective variants. In this latter case, the identification of the NRB binding site may potentially lead to the development of species-selective vasoconstrictors for use as species-selective, eco-sustainable pesticides or, alternatively, as selective vascular modulators for use in humans.

In the attempt to identify the cellular location of the NRB binding site we have recently developed a nitrobenzoxadiazole (NBD) derivative of NRB (NRB-AF12), and investigated its distribution in both NRB-sensitive cells, i.e., freshly isolated rat caudal artery myocytes (FIRCAMs) and NRB-insensitive cells (D'Amore et al., 2016). The results of our previous work revealed that in all cell types investigated NRB-AF12 clearly labeled intracellular structures such as endoplasmic reticulum (ER), Golgi apparatus, mitochondria and some lysosomes, without inducing any apparent cytotoxic effect. These findings suggested that an intracellular target could be involved in the vasoconstrictor effect of NRB and, at the same time, indicated NRB as a potential scaffold for the development of a new class of fluorescent probes for living cells (D'Amore et al., 2016). Compared to other fluorophores, NBD has the advantage of being relatively small in size, which theoretically reduces the risk of it inadvertently modifying the functional properties of the molecule to which it is conjugated. However, shortcomings include low quantum yields (that reduces the brightness of the fluorescent images) and limited photostability (resulting in the rapid bleaching of the probe on exposure to UV radiation). By contrast, boron dipyrromethene difluorides (BODIPYs), which constitute an extensive family of fluorescent dyes, display many improved photo-physical properties over their NBD counterparts, including larger absorption coefficients, higher fluorescence quantum yields, narrower emission profiles, improved photostability, lower sensitivity to changes in polarity and pH, and are also available in a broader range of colors (Karolin et al., 1994; Bergström et al., 2002; Ulrich et al., 2008). The use of fluorophores to label small molecules, in order to define their cellular distribution (and the potential site/s of action), is not without risk since the distribution of the fluorescent signal may not necessarily reflect that of the unlabeled compound itself. However, this risk may be reasonably mitigated by establishing that different derivatives carrying distinct fluorophores have similar cellular distribution.

In this study, we investigated the biological properties of a BODIPY FL-conjugated NRB derivative (MC009), and confirmed that (i) it had the same distribution profile as NBD-derived NRB-AF12 in NRB-contracted cells; (ii) it showed an improved fluorescent profile compared to NRB-AF12 with no apparent cytotoxic effects, indicating NRB as a suitable scaffold for the development of novel fluorescent probes for the labeling of cellular structures in both living and fixed cells.

## Materials and methods

### Ethics statement

The procedures involving animals were approved by the Animal Care and Use Ethics Committee of the University of Padova under license from the Italian Ministry of Health (project N°650/2015), and are in compliance with the National and European guidelines for handling and use of experimental animals.

### Chemistry

**Figure 1:**
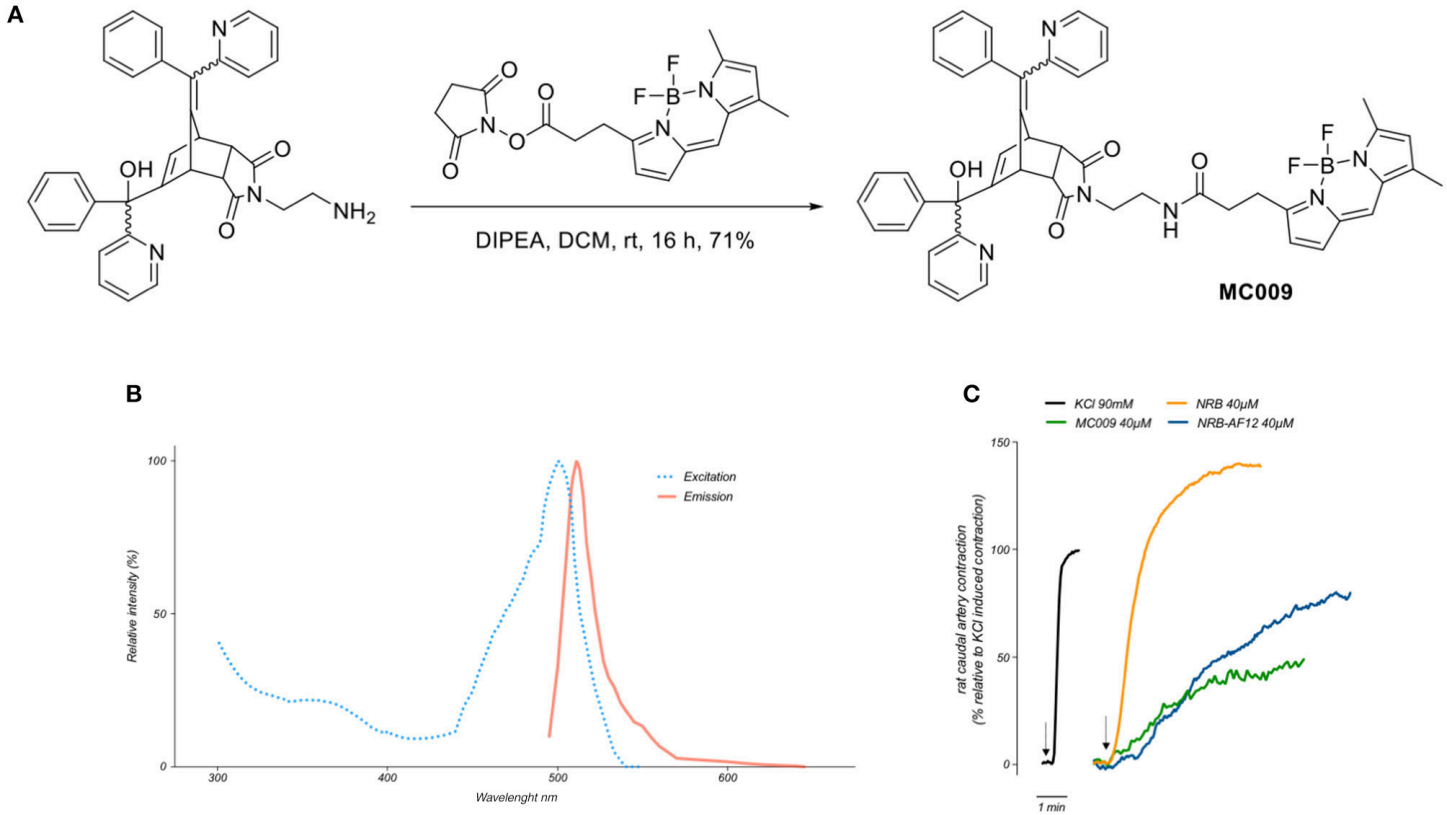
Synthesis, fluorescent spectrum and biological properties of MC009. **(A)** Scheme illustrating the synthesis of MC009. **(B)** Excitation and emission spectra of MC009. **(C)** Original records showing the contractile effects of KCl 90 mM, NRB (endo isomers) 40 μM, MC009 40 μM, and NRB-AF12 40 μM, in rat caudal artery rings.

The synthesis of MC009 is schematically represented in Figure 1A. BODIPY FL (3-[4,4-difluoro-5,7-dimethyl-4-bora-3a,4a-diaza-s-indacene-3-yl]propionic acid) (Gießler et al., 2010), along with its corresponding N-hydroxysuccinimide ester, BODIPY FL NHS ester (Sorkin et al., 2017), and N-2'-aminoethyl-endo-5-(α-hydroxy-α-2-pyridylbenzyl)-7-(α-2-pyridylbenzylidene)-5-norbornene-2,3-dicarboximide (Rennison et al., 2013) were prepared using literature methods. A solution of N-2'-aminoethyl-endo-5-(α-hydroxy-α-2-pyridylbenzyl)-7-(α-2-pyridylbenzylidene)-5-norbornene-2,3-dicarboximide (90 mg, 0.16 mmol), BODIPY FL NHS ester (69 mg, 0.18 mmol) and diisopropylethylamine (31 μL, 0.18 mmol) in dichloromethane (5 ml) was stirred at room temperature for 16 h. The mixture was washed with water, the separated aqueous phase further extracted with dichloromethane (2 × 10 ml), the combined organic layers dried over anhydrous magnesium sulfate, filtered and the solvent removed *in vacuo*. Purification by flash chromatography (petroleum ether/ethyl acetate, 1:2) afforded BODIPY FL-NRB conjugate MC009 as a mixture of endo stereoisomers (red solid; 100 mg, 0.12 mmol, 75%). ^1^H NMR (400 MHz, CDCl_3_) δ 2.22 (3H, s, CH_3_), 2.50 (3H, s, CH_3_), 2.51–2.58 (2H, m, NHCOC*H*_2_), 3.20–3.72 (8.2H, m, NHCOCH_2_C*H*_2_, NC*H*_2_C*H*_2_NHCO, H-2, H-3, W/H-4), 3.83–3.90 (0.8H, m, U/H-1, V/H-1, Y/H-4), 4.08 (0.1H, m, U/H-4), 4.29 (0.3H, m, V/H-4), 4.42–4.49 (0.6H, m, W/H-1, Y/H-1), 5.40 (br s, OH), 5.61–5.64 (0.7H, m, V/H-6, Y/H-6), 6.04–6.07 (1H, m, Ar), 6.19 (0.1H, m, U/H-6), 6.23 (0.2H, m, W/H-6), 6.26 (br s, OH), 6.27 (br s, OH), 6.29–6.31 (1H, m, Ar), 6.36 (m, NH), 6.47 (m, NH), 6.61 (m, NH), 6.75-7.60 (18H, m, = CH, Ar), 8.36-8.63 (2H, m, αPyr). m/z (ESI+, 70 eV) calcd for C_49_H_43_BF_2_N_6_NaO_4_ [M+Na]^+^: 851.3307; found: 851.3335. RP-HPLC: *t*_*R*_ = 12.5, 12.7 min (purity λ_254nm_ = >98%) An RF-HPLC chromatogram and ^1^H NMR spectrum are reported in Supplementary Figure 1).

Fluorescence spectra of MC009 (Figure 1B) was obtained by dissolving the compound in 100% DMSO to obtain a 2 mM stock solution, then diluted in Milli-Q water to reach the final concentration of 5 μM. Analysis of excitation/emission peaks were evaluated using a Jasco FP6500 spectrofluorometer (temperature 25°C; *b* = 1 cm; λ ex/em: 470/540; sli: 5/10 nm; data pitch 0.2 nm; scanning speed 200 nm/min).

### Cell culture

LX2 (a kind gift from Prof. S. Fiorucci, University of Perugia) cells were cultured at 37°C in an atmosphere of 5% CO_2_ in Dulbecco's Modified Eagle Medium (Euroclone-Milan, Italy) supplemented with 10% Fetal Bovine Serum (FBS) (Euroclone), 2 mM L-Glutamine and antibiotics (penicillin/streptomycin).

### Vascular smooth muscle cells (VSMCs) isolation

VSMCs were isolated from the tail main artery of male Wistar rats and C57BL/6 mice as previously reported (Fusi et al., 2002). Briefly, a 5-mm long piece of artery (rat) or the whole artery (mice) were incubated at 37°C for 40–45 min in 2 ml of 0.1 mM Ca^2+^ external solution containing 20 mM taurine, 1.35 mg/ml collagenase (type XI), 1 mg/ml soybean trypsin inhibitor, and 1 mg/ml BSA, which was lightly bubbled with a 95% O_2_ – 5% CO_2_ gas mixture to gently agitate the enzyme solution. Cells were stored in 0.1 mM Ca^2+^ external solution (NaCl 110 mM, KCl 5.6 mM, D-glucose 20 mM, taurine 20 mM, Hepes 10 mM, Na pyruvate 5 mM, MgCl_2_ 1.2 mM, BSA 0.5 mg/ml; pH 7.35) at 4°C under normal atmosphere for at least 1 h, and were used for experiments within 1 day following isolation; the same solution was used as incubation medium during the experiments involving these cells, as previously reported (Fusi et al., 2002).

### Live cell imaging

To define the cellular distribution of MC009 in living rats and mice VSMCs, cells were incubated with MC009 (100 nM) for 30 min in a humidified 5% CO_2_ incubator at 37°C. In separate experiments, cells were loaded with MC009 plus CellMask™ (1:5,000), a fluorescent probe for plasma membrane, or with the fluorescent Ca^2+^ indicator X-Rhod-1™ (1–2.5 μM). MC009 concentration (100 nM) and its loading protocol were chosen on the basis of preliminary experiments performed in LX2 cells, and is described in the results section. In living LX2 cells MC009 distribution was studied by loading it alone (10–100 nM) or in combination with fluorescent probes selective for intracellular structures: ER Tracker™ red (500 nM), a marker of endoplasmic reticulum, pHrodo™ (50 ng/ml), a marker of endosomes and lysosomes, LipidTOX™ (1:1,000), a marker of lipid droplets. Mitochondria ER distribution of MC009, were further investigated in LX2 cells transfected with COX-IV-RFP-pcDNA3.1 or calreticulin-RFP-pcDNA3.1 plasmids using Lipofectamine LTX reagent (Invitrogen), according to manufacturer's instructions. 24 h post transfection, cells were left untreated or primed with thapsigargin (5 μM) for 3 h to induce acute intracellular stress and swelling of the organelles, and finally loaded using MC009 (100 nM).

The distribution of MC009 was also investigated both during and after cell division. To this end, LX2 cells were chemically synchronized in the G2/M phase through exposure to RO-3306 (10 μM) for 18 h, with MC009 (1 μM) in the culture medium. Following treatment, cells were washed with reagent-free medium and the cell cycle was reinduced through the addition of 10% FBS. Cellular distribution of MC009 and cell division were monitored at 30 min intervals for 4 h.

### Immunocytochemistry

In order to evaluate MC009 co-localization with Golgi apparatus, 2 × 10^4^ LX2 cells were plated onto 12 mm coverslips and left to attach for 24 h. Cells were then fixed with 0.25% glutaraldehyde in 0.1 M sodium cacodylate, pH 7.4 for 5 min and permeabilized using 0.1% digitonin for 10 min; cells were incubated with GM130 antibody (1:500) for 90 min at room temperature. Following PBS washes, cells were fluorescently labeled with Cy3 goat anti-rabbit IgG fluorescent secondary antibody (1:500 for 60 min at room temperature) in the presence of MC009 100 nM. Finally, coverslips were mounted on microscope slides using Mowiol 40–88 (Sigma Aldrich) and images acquired under confocal microscope. In separate experiments cells were fixed using ice-cold methanol instead of glutaraldehyde.

### Contraction of rat caudal artery rings

The contractile activity of MC009 was evaluated in rings obtained from the ventral caudal artery of Wistar rats as previously described (Bova et al., 2001). Briefly, the rings, cleaned of the adventitia, were mounted in a home-made myograph by inserting two 100 μm thick tungsten wires into their lumen and immersed in 15 ml double-jacketed organ baths containing a salt solution of the following composition: NaCl 125 mM, KCl 5 mM, MgSO_4_ 1 mM, KH_2_PO_4_ 1.2 mM, CaCl_2_ 2.7 mM, NaHCO_3_ 25 mM and glucose 10 mM, bubbled with a mixture of O_2_ (95%) and CO_2_ (5%) and maintained at 37°C at a pH of 7.35. The contractile force was measured through an isometric force transducer (2B Instruments, Milano, Italy) coupled to an analog-to-digital converter (PowerLab). At the beginning of the experiment, a preload of 3 g (1 g/mm) was applied to each ring. The preloaded rings were left to equilibrate for at least 60 min and then repeatedly stimulated with 90 mM KCl and 10 μM phenylephrine until two consecutive reproducible contractile responses to each stimulus were obtained. The experiments were conducted in rings mechanically deprived of the endothelium; the absence of a functional endothelium was confirmed by the lack of carbachol-induced relaxation of rings pre-contracted with phenylephrine.

### MC009 cellular uptake

To evaluate the effect of endocytosis on MC009 uptake, LX2 cells were plated onto 12 mm coverslips and left to attach for 24 h. The cells were maintained at 4°C for 30 min to inhibit endocytosis and then treated with 80 μM Dynasore (an inhibitor of dynamin-dependent endocytosis) (Kirchhausen et al., 2008) for 30 min at 37°C, after which MC009 (100 nM) was added to the culture medium for an additional 30 min. pHrodo red transferrin conjugate (25 μg/ml), which is known to enter the cells via dynamin-dependent, dynasore-sensitive endocytosis, was used under the same conditions as a positive control. At the end of the incubation period cells were washed with PBS, rinsed with probe-free medium and immediately observed under confocal microscopy using a 40 × objective. Quantification of fluorescence was performed with Fiji software using the following formula: Corrected Total Cell Fluorescence (CTCF) = Integrated density - (Cell area x Mean of fluorescence of background readings) and reported as percentage relative to the CTRL (McCloy et al., 2014).

### MTT assay

LX2 cells were seeded (1.5 × 10^3^ cells/well) in 96-well tissue plates and exposed to increasing concentrations of MC009 (0.01, 0.1, 1, 10, and 50 μM) for 24 h. Cellular growth was evaluated by MTT reduction assay (Mosmann, 1983; Di Francesco et al., 2015). Briefly, at the end of the experiment, 20 μl of a sterile solution of tetrazolium salt (5 mg/ml in PBS) were added to each well, and following 4 h at 37°C the supernatant was discarded and the resultant formazan crystals were dissolved in 200 μl of dimethyl sulfoxide. Absorbance intensity was measured at 570 nm on a multi-well plate spectrophotometer (VICTORTMX3 2030 Multilabel Reader, Perkin Elmer).

### Confocal microscopy

Fluorescent images were collected using a Zeiss Axio Observer Z1 inverted confocal microscope equipped with a Plan-Apochromat 63 × /1.40 Oil DIC objective. For live imaging experiments, cells were maintained in an atmosphere of 5% CO_2_ and warmed at 37°C in a heated chamber.

### Sources of fluorescent probes, antibodies and chemicals

CellMask™ (#C10045), X-Rhod-1™ (#X14120), ER Tracker™ red (#E34250), pHrodo™ (#P10361), LipidTOX™ (#H34477) and pHrodo red transferrin conjugate (#P35376) were obtained from Thermo Fisher Scientific. Dynasore and Ro-3306 were purchased from Abcam, Lipofectamine LTX reagent from Invitrogen, Anti-GM130 antibody (#610822) from BD Biosciences and Cy3 goat anti-rabbit IgG fluorescent secondary antibody (#111-165-003) from Jackson ImmunoResearch.

### Hazards related to the use of NRB and its derivatives for research purposes

In HSNO (Hazardous Substances and New Organisms) terms, a substance is considered hazardous if it triggers any one of the threshold levels for any of the hazardous properties listed in the USA Environmental Protection Agency (EPA) guidelines, one of which is toxicity. NRB is extremely toxic to rats and as such is classified as an “extremely hazardous chemical” in the US and elsewhere. However, one of the key features of NRB is that its toxic activity is uniquely restricted to rats, having little or no activity in any other species tested (including humans). In fact, NRB's lethal activity is even further restricted as it seems only to be toxic to species within the Rattus genus, with other rat genera being little or not affected. Consequently, both NRB and the NRB derivatives reported in this research paper pose little or no hazard to the researchers, and as such no special conditions or safety precautions are required to work with these substances other than those required to conform to standard laboratory practices.

### Statistical analysis

All values are expressed as means ± standard error mean (SEM) of *n* observations/group. Analysis of all the images and the computation of the Pearson's coefficients of co-localization were performed using the open-source platform for biological-image analysis Fiji software. Comparison of two groups was obtained by the Student's *t*-test for unpaired data where appropriate. Comparison of more than two groups were made with one-way ANOVA using *post-hoc* Tukey's test. Differences were considered statistically significant at values of *P* < 0.05.

## Results

### MC009 fluorescence spectrum and vasoconstrictor activity

By analysing the excitation and emission peaks at 500 and 511 nm, respectively, we were able to confirm that the fluorescent spectrum of MC009 overlapped with that of the parent BODIPY FL fluorophore (Figure 1B).

Given MC009 is a BODIPY-conjugate of the endo (contracting) isomers of NRB, we tested its effect on rat caudal artery rings (RCAr) to verify if the compound retained the vasoconstrictor properties of the parent NRB. As reported in Figure 1C, MC009 contracted RCAr, although with less effectiveness when compared to NRB itself, with the contractile effects of NRB and MC009 (expressed as percent of the 90 mM KCl-induced contraction) being 140 and 50%, respectively. NBD-derived NRB-AF12 likewise showed a contractile effect in RCAr, with an effectiveness equivalent to 70% of that induced by KCl (Figure 1C). A concentration of 40 μM was chosen for both compounds in these experiments because at higher concentrations MC009 precipitated from the incubation medium.

### MC009 loading procedures in living and fixed LX2 cells

**Figure 2:**
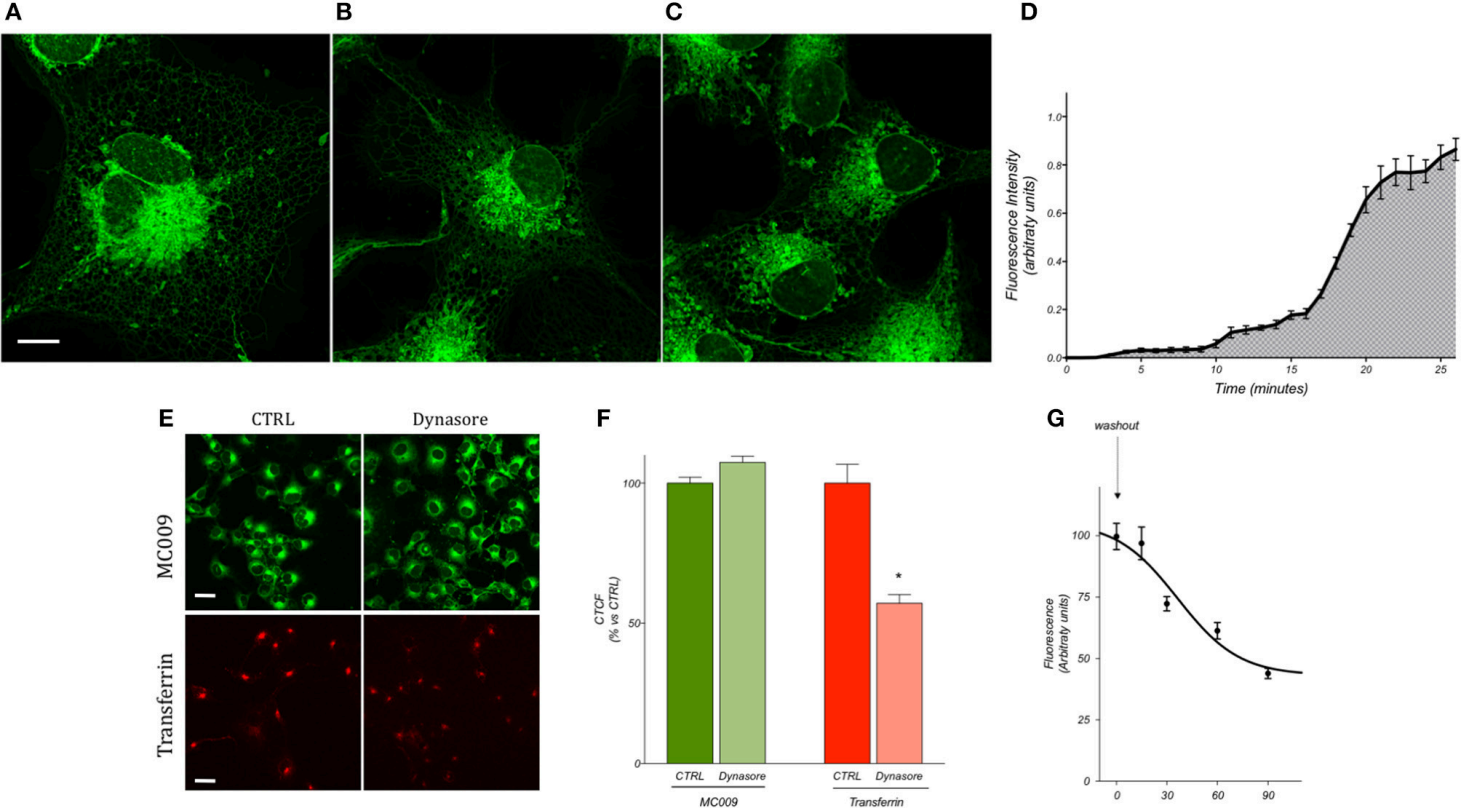
MC009 distribution and kinetics of internalization in living LX2 cells. Confocal live imaging of LX2 cells labeled with MC009 **(A)** 100 nM, **(B)** 50 nM, and **(C)** 10 nM, showing subcellular distribution of the probe. Magnification 63 × ; scale bar 10 μm. **(D)** MC009 internalization in LX2; data reported was extrapolated from a time-lapse of LX2 cells exposed to MC009 100 nM and imaged every 30 s at a single focal plan for about 30 min. **(E)** Effects of dynasore on the internalization of MC009 or pHrodo Red Transferrin-conjugate (used as positive control). Magnification 40 × ; scale bar 20 μm. **(F)** Quantification of fluorescent signal reported as percentage of CTRL. Data reported represent mean ± SEM; ^*^p < 0.05 vs. CTRL; *n* ≥ 150 cells. **(G)** LX2 cells were incubated for 30 min with MC009; after replacing the staining solution with probe-free medium, evaluation of MC009 extent of fluorescent signal was performed.

Preliminary experiments were carried out to establish the most optimum experimental parameters (with regard to both compound concentration and loading procedure) which would facilitate the best visualization of MC009 intracellular fluorescence distribution in living cells. The experiments were performed in human cultured hepatic stellate cells (LX2 cells), the same cell line that was previously used to characterize the intracellular fluorescence distribution of NBD-derivative NRB-AF12 (D'Amore et al., 2016). The results indicated that the best defined images of the intracellular organelles could be attained by incubating the cells with 100 nM MC009 for 30 min at 37°C (Figure 2A); however, an acceptable fluorescent signal could also be achieved using lower concentrations of the probe, i.e., 50 nM (Figure 2B) and 10 nM (Figure 2C). MC009 concentrations higher than 100 nM did not lead to a significant improvement in image quality (data not shown). The time-course of the cellular uptake of MC009 was characterized by collecting a single focal plan video of LX2 cells exposed to 100 nM MC009 (Supplementary Video 1). The results, summarized in Figure 2D, indicate that following the addition of the fluorescent dye to the incubation medium, the labeling of intracellular structures was detectable within 60 s, was clearly evident within 5 min and reached a plateau at 30 min. Higher concentrations (>100 nM) and/or longer incubation times did not significantly ameliorate the quality of the fluorescent images (data not shown). MC009 uptake was not affected by dynasore, an inhibitor of dynamin-dependent endocytosis, suggesting that its entry is not related to endocytotic processing; in contrast, under the same experimental conditions, dynasore significantly reduced transferrin uptake, which is known to be a dynamin-dependent process (Figures 2E,F). We also investigated the retention time of MC009, evaluated as fluorescence intensity, in LX2 cells loaded with the probe (100 nM for 30 min) and then incubated in a probe-free medium and imaged every 15–30 min. The results, summarized in Figure 2G, indicate that a clear staining of intracellular organelles could be detected for at least 90 min following removal of the fluorescent dye from the incubation medium.

Images of fixed LX2 cells incubated with 100 nM MC009 were comparable to those obtained in living cells (Supplementary Figure 1A), although the fluorescence signal was not well retained post-permeabilization and was significantly reduced due to the numerous washings required during the immunocytochemistry experimental procedure. The comparison of different fixation-permeabilization protocols indicated that the best results could be obtained using cross-linking fixative reagents (i.e., 0.25% glutaraldehyde) that preserve cell structures; in contrast, fixation of cells with methanol, which in addition to precipitating proteins removes lipids from the cells, resulted in low-defined images of intracellular structures by MC009 (Supplementary Figures 2A,B).

### MC009 distribution in LX2 cells

**Figure 3:**
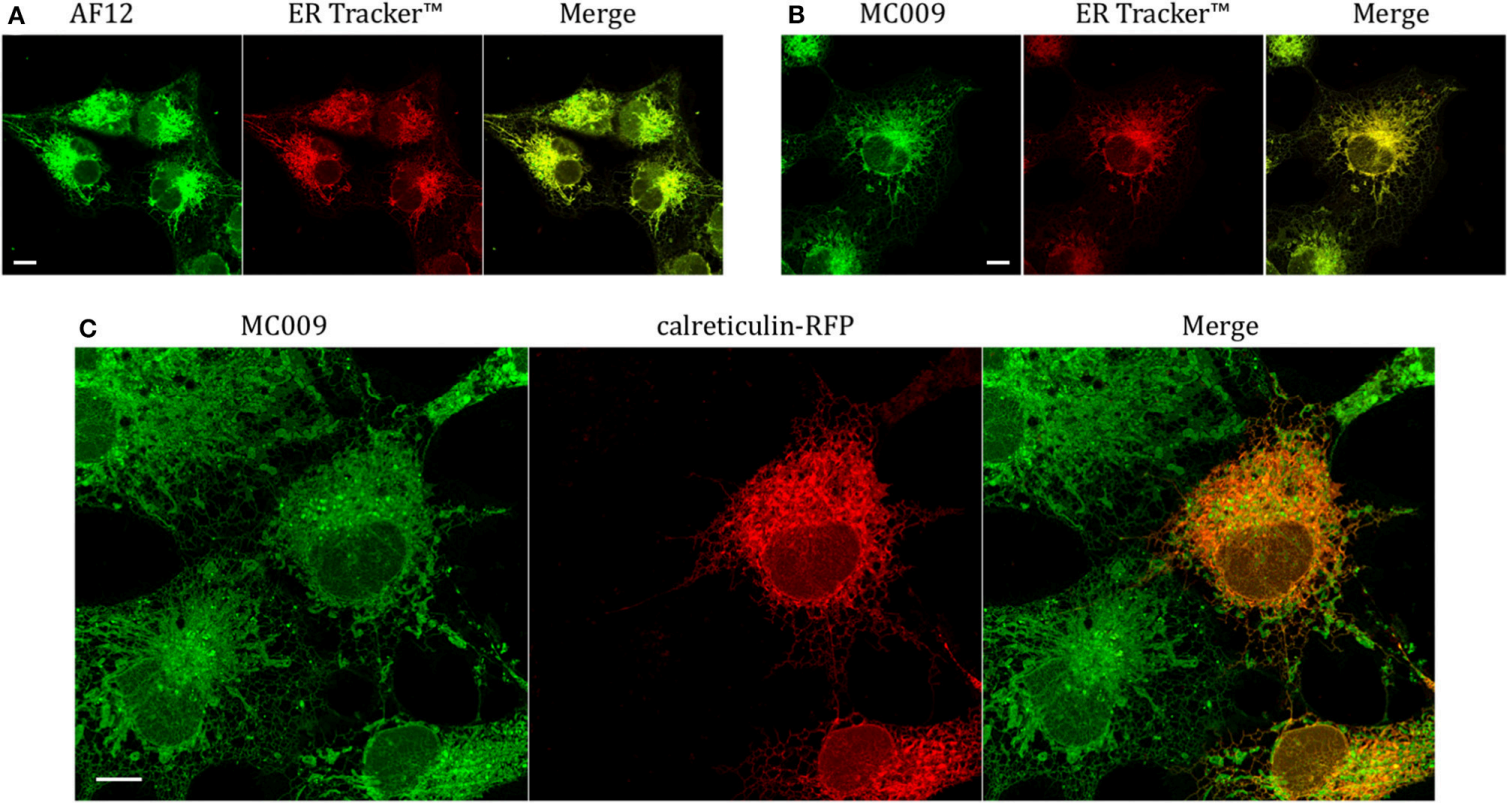
Endoplasmic reticulum markers and MC009 co-localization in living LX2 cells. Representative live cells microphotographs showing co-localization between **(A)** NRB-AF12 and **(B)** MC009 with ER-Tracker^TM^ in LX2 cells. **(C)** Live cell imaging of liver hepatic stellate cells transfected with the ER marker calreticulin-RFP and then stained using MC009. For each image, merged fluorescence is shown in right panel. Magnification 63 × ; scale bar 10 μm.

In order to establish if the conjugation of different fluorophores to NRB could influence its cellular distribution, we compared the distribution of MC009 and NRB-AF12 in LX2 cells. Since the two fluorescent dyes have not widely differing excitation/emission spectra (NRB-AF12 ex/em: 470/540 D'Amore et al., 2016; BODIPY FL ex/em: 502/511), a direct co-localization study was not possible. As a consequence, we used ER-Tracker™ red as an NRB-AF12 surrogate based on the finding that NRB-AF12 and ER Tracker™ red have identical distribution intracellularly (D'Amore et al., 2016). Our results, reported in Figures 3A,B and Tables 1, 2 clearly show that both NRB-AF12 and MC009 fluorescence overlapped with that of ER-tracker, strongly supporting the idea that, despite bearing different fluorophores, MC009 and NRB-AF12 label the same intracellular structures. Of note is the lack of plasma membrane staining by both MC009 and NRB-AF12, and a well-defined localization of both dyes to intracellular structures that have been previously identified as targets for NRB-AF12, e.g., ER, mitochondria, Golgi apparatus endosomes and lysosomes (D'Amore et al., 2016). To confirm that MC009 and NRB-AF12 bound to the same intracellular organelles, MC009 was used in combination with various fluorescent probes and/or antibodies that specifically target the NRB-AF12-labeled intracellular structures. The distribution of MC009 to ER was evaluated by transiently transfecting LX2 cells with a plasmid expressing the luminal ER resident protein calreticulin-RFP, and then loading the cells with the fluorescent dye. The results, reported in Figure 3C and in Table 1, show that MC009 fluorescent signaling significantly overlaps with that of calreticulin-RFP (Pearson's Coefficient: 0.65 ± 0.09; *n* ≥ 10). The binding of MC009 to ER did not apparently affect its organization and continuous remodeling, as shown in the Supplementary Video 2.

**Table 1:** Computation of Pearson's co-localization coefficient between MC009 and fluorescent probes/protein/antibodies targeted to specific subcellular structures (i.e., ER Tracker™, COX-IV-RFP, calreticulin-RFP, α-GM130, pHrodo™, LipidTOX™, Cell Mask™, and X-Rhod 1™).

**MC009**	**Pearson's Coeffficient (mean ± SEM)**
ER Tracker^TM^	0.96 ± 0.03
COX IV_RFP	0.46 ± 0.14
Calreticulin_RFP	0.65 ± 0.09
GM130	0.31 ± 0.07
Lipid TOX^TM^	0.22 ± 0.08
pHrodo^TM^	0.08 ± 0.04
CellMask^TM^	−0.07 ± 0.1
X-Rhod 1^TM^	0.69 ± 0.15

*All values are expressed as means ± SEM; n ≥ 10*.

**Table 2:** Quantification of NRB-AF12 and ER-Tr co-localization is shown as Pearson's coefficient; data are reported as means ± SEM; *n* ≥ 10.

**AF12**	**Pearson's Coeffficient (mean ± SEM)**
ER Tracker^TM^	0.95 ± 0.02

**Figure 4:**
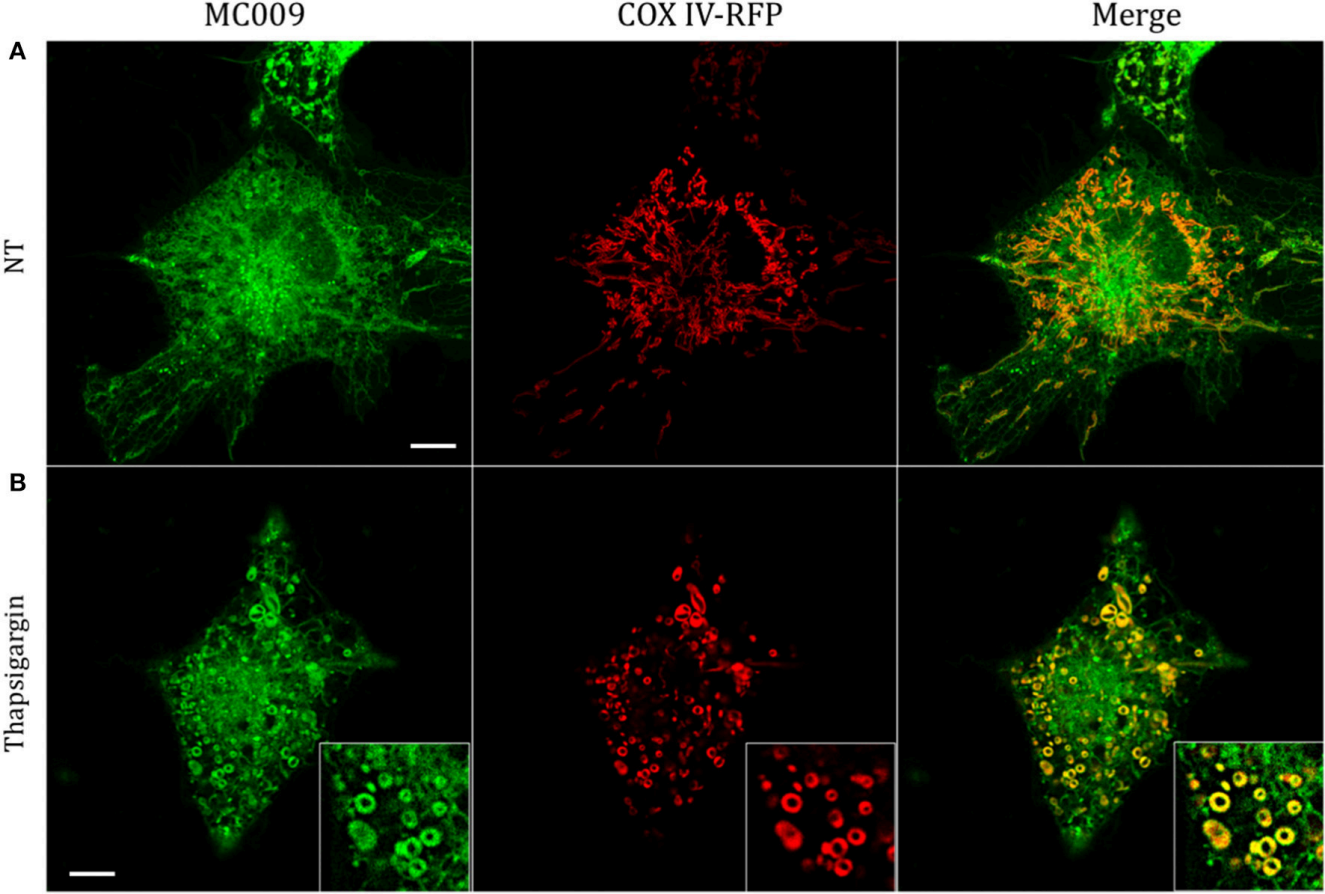
Mitochondrial markers and MC009 co-localization in living LX2 cells. Confocal live cell imaging of LX2 cells transfected with COX-IV-RFP plasmid and subsequently stained with MC009; cells were **(A)** left untreated or **(B)** primed with thapsigargin. Insets show magnification of the pictures. Magnification 63 × ; scale bar 10 μm.

**Figure 5:**
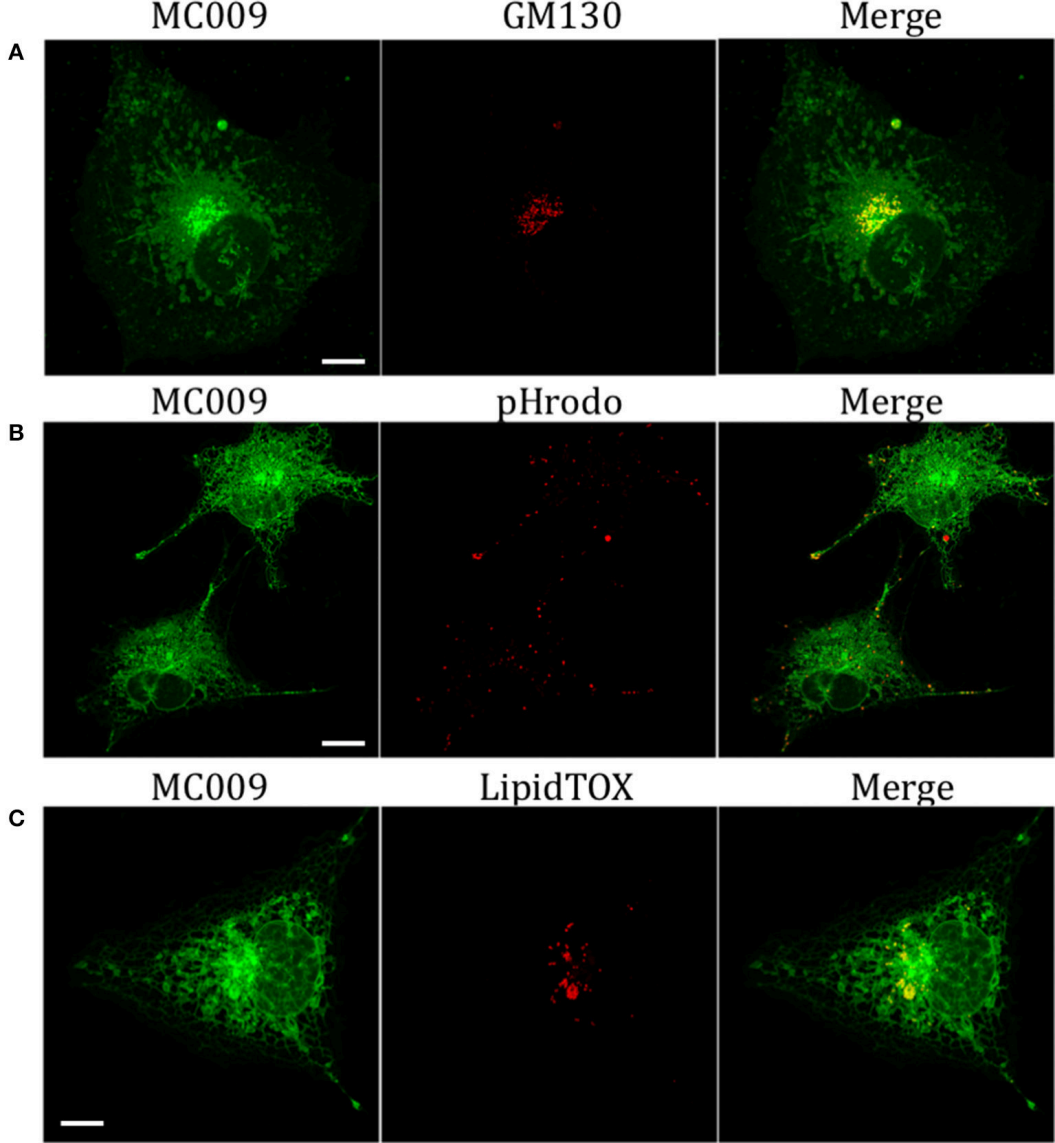
Golgi, endosomes, lysosomes and lipid droplets markers co-localization with MC009. **(A)** LX2 fixed cells immunostained for the Golgi apparatus structural protein GM130; cells were counter-labeled with MC009. **(B,C)** MC009 labeled LX2 cells counterstained with **(B)** the fluorescent dye for endosomal and lysosomal compartments or **(C)** for lipid droplets. Magnification 63 × ; scale bar 10 μm.

The potential for mitochondrial localization of MC009 was investigated in living LX2 cells transiently transfected with COX-IV-RFP plasmid to visualize the mitochondrial membrane resident protein COX-IV. As depicted in Figure 4A, and as reported in the computation of the Pearson's index of co-localization (Table 1), MC009 clearly labeled mitochondria. To assess whether MC009 bound the mitochondrial membrane, COX-IV-RFP transiently transfected cells were treated with thapsigargin to induce mitochondrial swelling (Korge and Weiss, 1999). Confocal images indicate a strong labeling of the mitochondrial membrane by MC009 (Figure 4B), a result that was confirmed in LX2 fixed cells labeled with MC009 and immunostained for COX-IV (Supplementary Figure 3). To verify whether MC009 bound the Golgi apparatus, LX2 fixed cells were immunostained with antibodies directed against the Golgi structural protein GM130 (α-GM130). The results, shown in Figure 5A, indicate a co-localization of MC009 and α-GM130 (Pearson's coefficient: 0.31 ± 0.07; *n* ≥ 10), confirming that MC009 partially overlapped with Golgi apparatus markers. Binding to the vesicles of endocytic pathway (i.e., endosomes and lysosomes) was investigated by co-staining LX2 cells with MC009 and the commercially available fluorescent probe pHrodo^TM^, which has been previously demonstrated to stain organelles involved in endocytosis (D'Amore et al., 2016). As shown in Figure 5B, there was in general no co-localization between the two fluorescent dyes (Pearson's index: 0.08 ± 0.04); however, a deeper analysis of the confocal images revealed that a few vesicles were positive for MC009 staining.

Finally, we observed a localization of MC009 in spherical particles that were identified, by incubation with the fluorescent probe LipidTOX™, as lipid droplets, i.e., the intracellular sites for neutral lipid storage (Figure 5C).

### MC009 toxicology in LX2 cells

**Figure 6:**
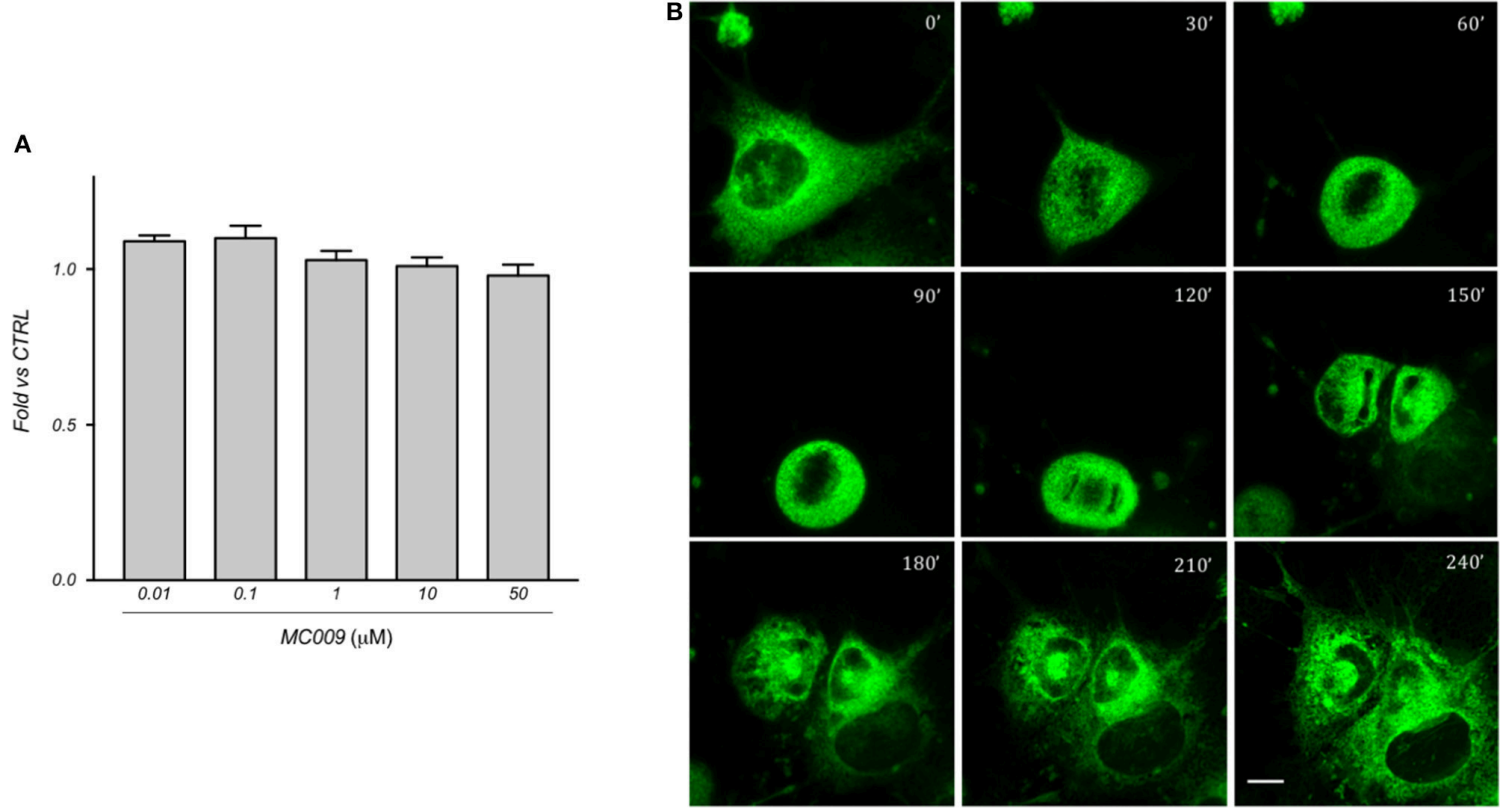
MC009 biocompatibility in LX2 cells**. (A)** Biocompatibility of MC009 was evaluated by MTT reduction assay. LX2 cells were treated for 24 h with increasing doses (0.01–50 μM) of MC009. Results are relative to untreated cells, that were arbitrarily set to 1. Data reported represent the mean ± SEM of three independent experiments. Statistical analysis of the MTT data by one-way ANOVA revealed no-significative differences between all groups. **(B)** MC009 biocompatibility with cell cycle progression. LX2 cells, chemically synchronized in G2/M by RO-3306, were imaged by confocal microscope after the re-induction of cell division by exposure to 10% FBS; images were acquired every 30 min at a single focal plan for 4 h. Magnification 63 × ; scale bar 10 μm.

The cytotoxicity of MC009 was assessed by priming LX2 cells with increasing doses (ranging from 10 nM to 50 μM) over a 24 h period; results obtained from MTT assays revealed that the fluorescent dye is non-toxic to the cells, even at concentrations much higher than those needed for cell staining (Figure 6A).

In addition, we investigated the distribution of MC009 during mitosis in LX2 cells. To this end, cells were chemically synchronized at the G2/M transition by exposing them to 10 μM RO-3306, an inhibitor of cyclin-dependent kinase 1 activity, and then loaded with MC009. Mitosis and cell cycle was then restored by incubating cells with RO-3306-free medium supplemented with FBS 10%, and the intracellular distribution of fluorescence was monitored for 4 h. In mitotic LX2 cells, MC009 distribution was confined to the perinuclear region, the majority of the signal being excluded from the spindle region, whereas a fraction of the MC009 fluorescence was visualized close to the spindle poles. As shown in Figure 6B, MC009 is transferred to daughter cells and a gradual recovery of a reticular appearance occurs following cellular division.

### MC009 distribution in FIRCAMs

**Figure 7:**
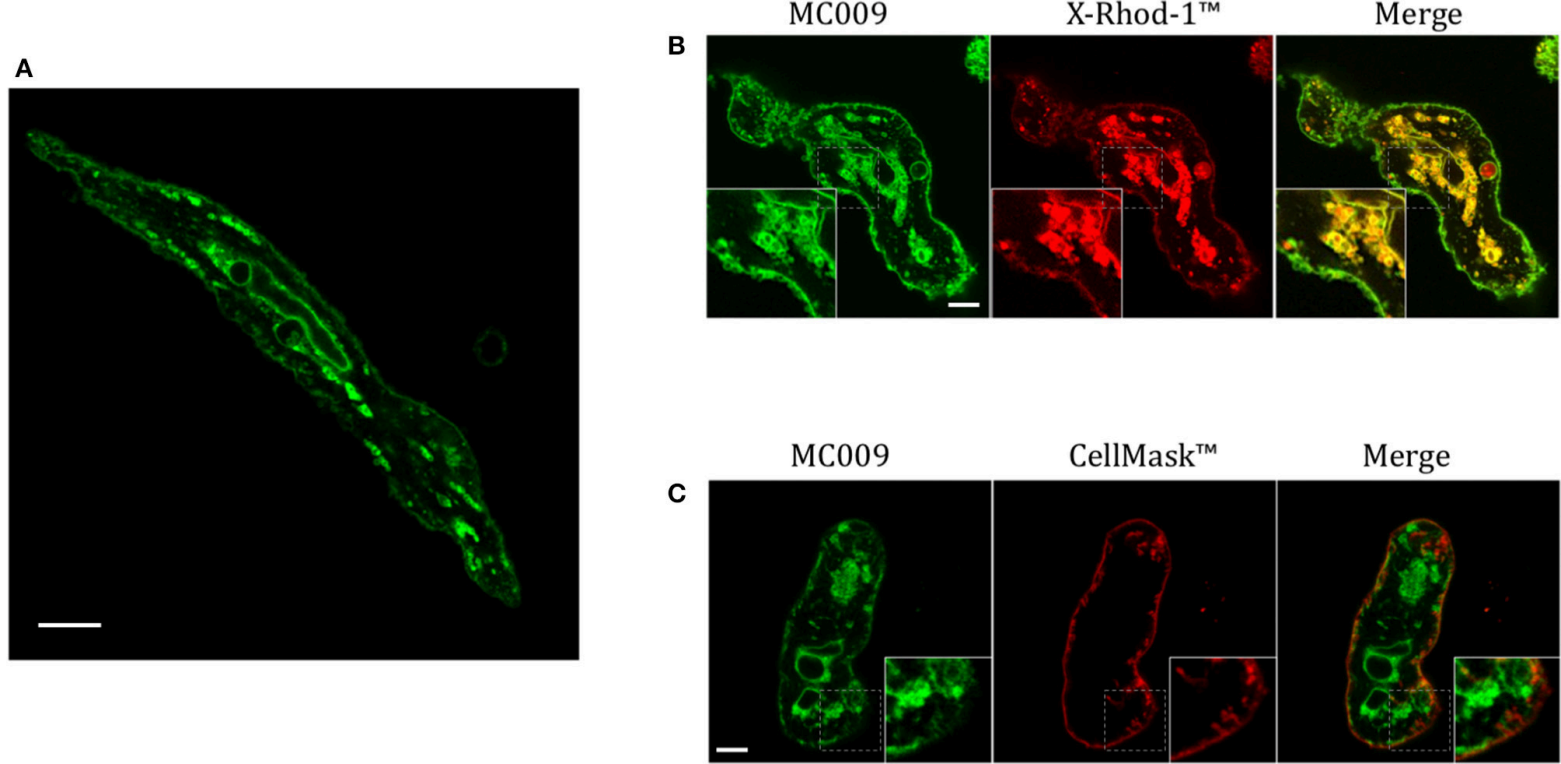
MC009 distribution in FIRCAMs. **(A)** Confocal live imaging of a FIRCAM fluorescently labeled with MC009 100 nM. In live imaging of rat primary vascular smooth muscle cells labeled with MC009 and counterstained with **(B)** the calcium indicator X-Rhod-1™ or **(C)** the florescent probe for plasma membrane CellMask™. Insets show magnification of the pictures. Magnification 63 × ; scale bar 5 μm.

Figure 7A is an image of a FIRCAM loaded with MC009. FIRCAMs represent the major cell type that are sensitive to NRB, responding to NRB application with a rapid and irreversible contraction (Fusi et al., 2002). The image highlights a clear labeling of intracellular structures with no apparent localization in the cytoplasmic milieu (Figure 7A and Supplementary Video 3). The fluorescent structures consist of some central elements and a sub-plasmalemmal network that coincide with intracellular calcium stores, as visualized by counterstaining the cell with the low-affinity calcium indicator X-Rhod-1™ (Figure 7B) or CellMask™ (Figure 7C), a fluorescent probe used for the staining of plasma membrane. The figure also shows that MC009 fluorescence distribution strongly co-localizes with that of X-Rhod-1 (Pearson's coefficient: 0.69 ± 0.15), but not with that of CellMask™ (Pearson's coefficient: −0.07 ± 0.1), revealing that MC009 distribution in these cells replicates that observed in LX2 cells. Quite similar results were obtained in VSMCs isolated from mouse caudal artery, which are insensitive to NRB (Supplementary Figure 4).

## Discussion

### MC009 as a new prototype fluorescent tool for visualizing intracellular structures in living cells

The use of fluorescent probes for live cell imaging represents an important technique to observe and monitor a plethora of biological processes, such as morphological cellular changes, activity and remodeling of intracellular structures, membrane trafficking and vesicle formation. Being able to conduct such studies on living cells avoids the artifacts or cell alterations that can often occur during the cell fixation process. One of the main advantages of live cell imaging is the capacity to dynamically observe over time all of these biological processes in a non-invasive manner, without alteration or interference in any way, provided that the probes are endowed with good photostability, long-term tracing, high signal-to-noise ratio, and a lack of cell toxicity.

In this study we show that conjugation of the BODIPY FL fluorophore to NRB afforded a compound (MC009) endowed with excellent fluorescent properties, coupled with a lack of cytotoxicity, that make it a suitable tool for living cell imaging. Compared to NRB-AF-12, an NBD-derived NRB fluorescent probe, MC009 shows lower photo-bleaching, better stability of the fluorescent signal, and better demarcation of the labeled structures obtained at concentrations lower than those required for NRB-AF12 (10–100 nM vs. 500 nM). MC009 cellular internalization was relatively fast and apparently not mediated by endocytosis, allowing an optimal staining of intracellular organelles following a few minutes of incubation. Moreover, a bright fluorescent signal of MC009 was retained for at least 90 min following its removal from the culture medium, and it is plausible that this signal could be retained much longer after washout, enabling more extended observations to be possible (Figure 2). In living LX2 cells MC009 allows a clear visualization of the ER network, mitochondria and the Golgi apparatus, likely as a consequence of a greater concentration of the fluorescent probe in these structures, in which it seems to target the membranes rather than to accumulate inside the lumen (Figures 4B, 7B). Of note, MC009 did not apparently disturb the physiological dynamic of either ER or mitochondria (Supplementary Video 2), as there were no alterations observed in their morphology (Figures 3, 4), indicating that the fluorescent dye is well tolerated by the cells. As previously reported for NRB-AF12 (D'Amore et al., 2016), MC009 also demonstrated a great biocompatibility, showing no signs of cellular toxicity, as detected by cell viability tests, even when the cells were exposed for 24 h at concentrations as high as 50 μM. In addition, MC009 did not impair the regular progression of cell division (Figure 6B), allowing a detection of the ER and mitochondria dynamics during mitosis. Another interesting feature of MC009 is the possibility to label cellular structures in fixed cells. In this regard, we found that the best results could be obtained by using cross-linking fixative reagents, which allowed us to obtain confocal images comparable with those obtained in living cells (Figure 2 and Supplementary Figure 2).

Given the intracellular distribution of the two NRB fluorescent conjugates used in this study remained unaffected on changing the fluorophore, it is reasonable to assume that the parent NRB could be readily modified through conjugation with other fluorophores, such as those belonging to the boron-dipyrromethene difluoride family that have different excitation/emission spectra (i.e., BODIPY TMR, BODIPY TR and others), without altering its labeling properties. If achievable, this would enable the creation of a new class of NRB-derived fluorescent dyes, excitable at different wavelengths to those currently available, consequently expanding the range of probes that can be used for live cell imaging. In addition, MC009 did not interfere with other dyes (i.e., ER Tracker™, LipidTOX™, or pHrodo™) when used simultaneously for *living* cell staining of LX2 cells, excepted for the co-incubation with MitoTracker™, which resulted in mitochondria toxicity as demonstrated by a rapid swelling of the organelles (data not shown).

### MC009 as a tool to identify the cellular target of NRB in FIRCAMs

The results of this study show that in living FIRCAMs MC009 targets the membranes of intracellular structures but does not bind to the plasma membrane (Figure 3), as verified by the lack of co-localization of MC009 and CellMask™ (Figure 7C). In particular, we show a clear binding of MC009 to sarcoplasmic reticulum (SR) and mitochondria, as previously reported for NRB-AF12 in the same cells (D'Amore et al., 2016), that leads to the conclusion that, in NRB-sensitive cells also, the distribution of the fluorescent derivatives of NRB is not affected by the type of fluorophore attached to the parent molecule. This finding, together with the observation that both NRB-AF12 and MC009 (present study) partly retain the capacity of the parent compound to induce contraction in rat caudal artery rings, supports the hypothesis that the distribution of the fluorescent NRB-conjugates reflects that of NRB itself, making it possible that the vasoconstriction activity of NRB could be mediated through an intracellular binding site.

The species-selectivity of the vasoconstrictor effect of NRB prompted us to investigate if the intracellular fluorescent distribution of MC009 observed in FIRCAMs could differ from that observed in the vascular myocyte of a NRB-insensitive species, i.e., the mice. As reported in Figure 7 and Supplementary Figure 4, there was apparently no difference in distribution between FIRCAMs and freshly isolated mice caudal artery myocytes. Furthermore, the fluorescence distribution of the corresponding exo isomers of MC009, which is not a vasoconstrictor, was found to be the same as that observed with the corresponding endo-specific MC009, as used in this study (S. Bova, personal communication). These results may indicate that both the endo and exo isomers of NRB bind to the same intracellular sites in all cell types; however, only in the rat vascular myocytes does this binding lead to a rise of cytoplasmic free calcium concentration and contraction. Another possibility is that MC009 distribution does not necessarily reflect that of the parent molecule. It has been reported that the parent BODIPY FL itself can bind to intracellular lipids, thus influencing the distribution of the molecule to which it is attached (Courtis et al., 2014). Such a scenario can be observed with the antidiabetic agent glibenclamide, which inhibits plasma membrane K_ATP_ channels targeting their SUR subunits (Proks et al., 2002): its BODIPY derivative, ER-Tracker™, does not label the plasma membrane, as expected (D'Amore et al., 2016). However, in many other cases, the fluorescent derivatives of small molecules show a cellular distribution that is in agreement with the binding sites of the unlabeled parent compounds, as in the case of thapsigargin (Shmygol and Wray, 2004; Gómez-Viquez et al., 2010), ryanodin (Gordienko et al., 2001; Shmygol and Wray, 2004; Gómez-Viquez et al., 2010), ceramide, brefaldine A (Deng et al., 1995; White and McGeown, 2002) and ouabain (Alonso et al., 2013). In the case of MC009, a couple of observations make this possibility unlikely: (1) the distribution of MC009 overlaps that of AF12, in which NRB is conjugated to an NBD group; (2) MC009 retains the biological activity of the parent molecule, and therefore it must bind to the NRB target.

## Conclusions

In conclusion, on the basis of the results of this study, we hypothesize the rat specific vasoconstrictor effect of NRB to be mediated by an intracellular target localized on the myocytes of rat peripheral arteries. In addition, we propose MC009 as a new, high performance, non-toxic fluorescent probe for the labeling of intracellular organelles in living cells; modification of the NRB scaffold, to create new fluorescent probes capable of selectively labeling different intracellular organelles, represents a stimulating perspective for the future.

## Author contributions

SB and CD conceived the study. MB, DR, BH, and MJ-S designed and performed the synthesis of MC009. CD, GO, AF, and GR designed and performed experiments. SB, CD, GC, and AF analyzed and interpreted data. SB, CD, DR, and BH wrote the manuscript. All authors approved the final version of the manuscript.

### Conflict of interest statement

The authors declare that the research was conducted in the absence of any commercial or financial relationships that could be construed as a potential conflict of interest.

## Supplementary material

Supplementary Figure 1 MC009 RF-HPLC chromatogram and ^1^H NMR spectrum**. (A)** Reverse-phase HPLC [Dionex P680 system using a Phenomenex Gemini C_18_-Si column (150 × 4.6 mm, 5 μm)] data for MC009—eluted using a gradient of 100:0% A/B to 5:95% A/B over 15 min at 1 mL/min; where solvent A was water (0.1% trifluoroacetic acid) and solvent B was CH_3_CN (0.1% trifluoroacetic acid); with detection at 254 nm. **(B)** ^1^H NMR spectrum acquired at 400 MHz in CDCl_3._

Supplementary Figure 2 MC009 distribution in LX2 fixed cells. LX2 cells stained with MC009 were fixed and imaged by confocal microscopy. **(A)** Glutaraldehyde fixed cells; **(B)** methanol fixed cells. Insets show magnification of the pictures. Magnification 63 × ; scale bar 10 μm.

Supplementary Figure 3 Mitochondrial markers and MC009 co-localization in LX2 fixed cells. LX2 fixed cells immunostained for the mitochondrial membrane protein COX-IV; cells were counter-labeled with MC009. Scale bar 10 μm.

Supplementary Figure 4 Distribution of MC009 in mouse vascular smooth muscle cells. Live imaging of mouse primary vascular smooth muscle cells labeled with MC009 and counterstained with the calcium indicator X-Rhod-1™. Magnification 63 × ; scale bar 10 μm.

Supplementary Video 1 Time-lapse recording of MC009 internalization in LX2 cells. Cells were exposed to MC009 (100 nM) and imaged every 30 s at a single focal plan for 25 min. Scale bar represents 10 μm.

Supplementary Video 2 MC009 labeling did not affect ER organization and continuous remodeling. Cells were exposed to MC009 (100 nM) and imaged every 5 s at a single focal plan for 3 min. Scale bar represents 3 μm.

Supplementary Video 3 3D reconstruction of a FIRCAM labeled with MC009. Magnification 63 × ; scale bar 5 μm.
