# Supplementary figures and images for: Synthesis and Biological Characterization of a New Norbormide Derived Bodipy FL-Conjugated Fluorescent Probe for Cell Imaging
Source: Front Pharmacol. 2018 Sep 25;9:1055. doi: 10.3389/fphar.2018.01055 (PMC6168047; doi:10.3389/fphar.2018.01055)

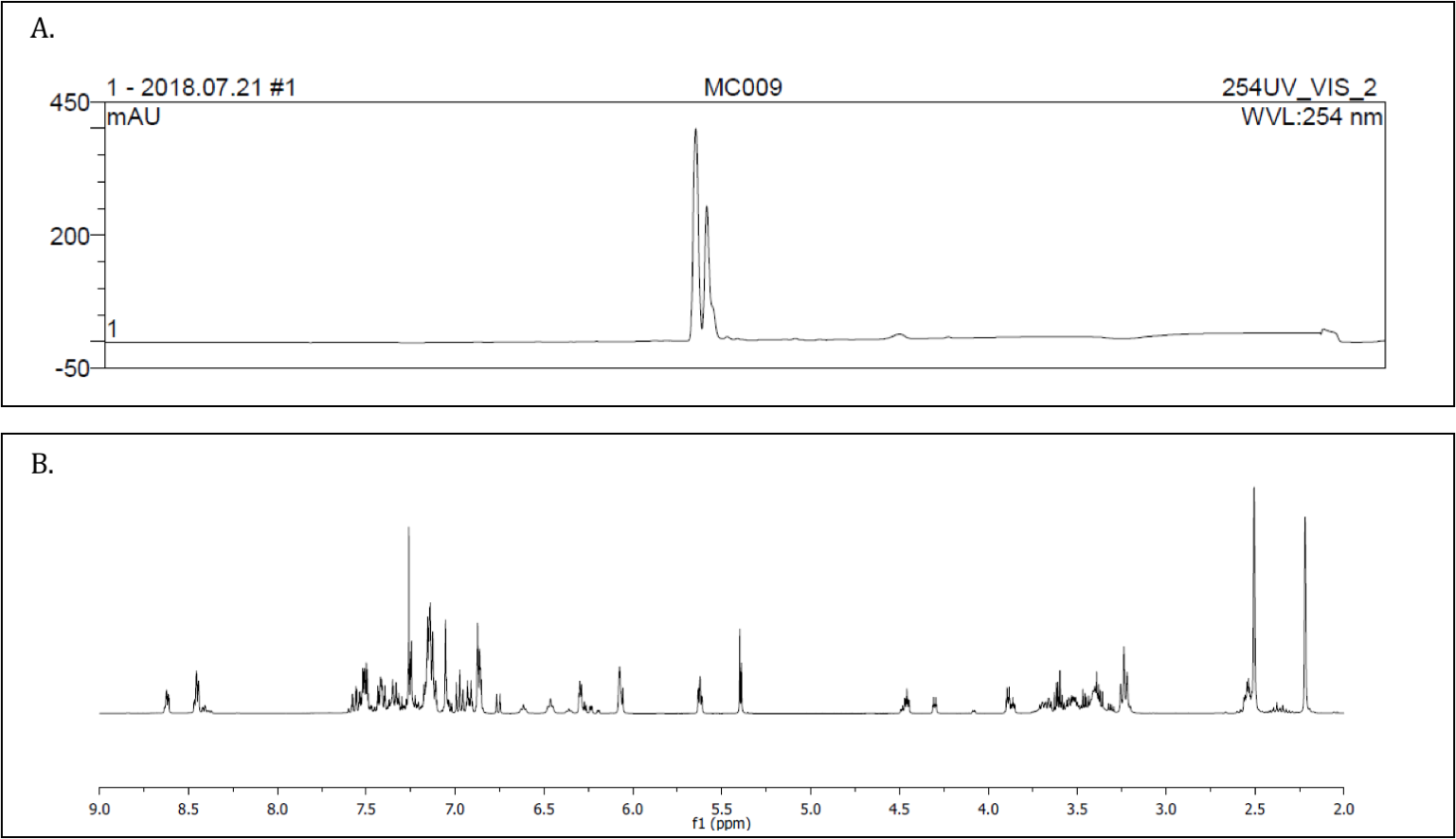

Supplement: Supplementary Figure 1 — MC009 RF-HPLC chromatogram and 1H NMR spectrum. (A) Reverse-phase HPLC [Dionex P680 system using a Phenomenex Gemini C18-Si column (150 × 4.6 mm, 5 μm)] data for MC009—eluted using a gradient of 100:0% A/B to 5:95% A/B over 15 min at 1 mL/min; where solvent A was water (0.1% trifluoroacetic acid) and solvent B was CH3CN (0.1% trifluoroacetic acid); with detection at 254 nm. (B) 1H NMR spectrum acquired at 400 MHz in CDCl3. [file Image_1.TIFF]

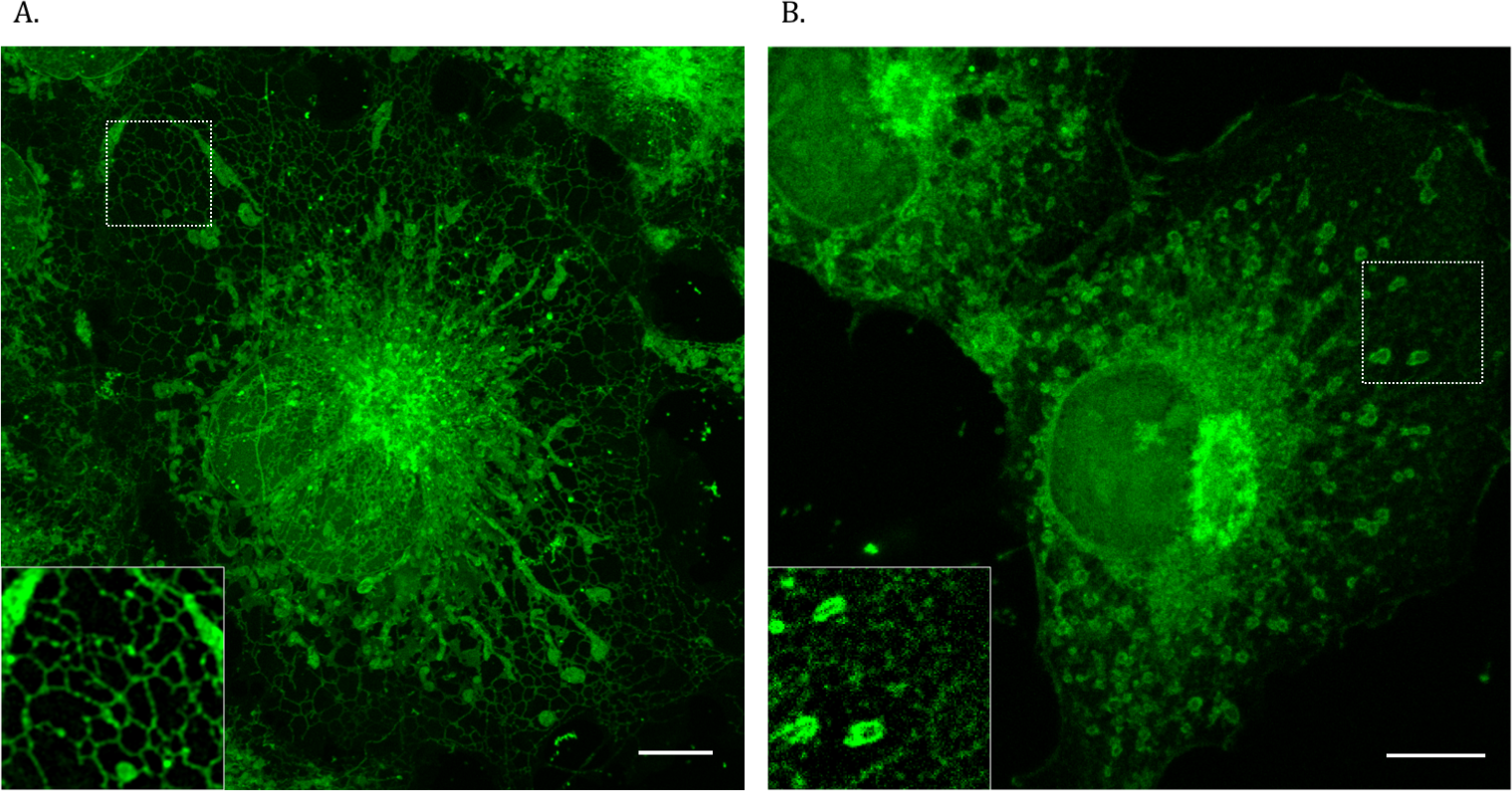

Supplement: Supplementary Figure 2 — MC009 distribution in LX2 fixed cells. LX2 cells stained with MC009 were fixed and imaged by confocal microscopy. (A) Glutaraldehyde fixed cells; (B) methanol fixed cells. Insets show magnification of the pictures. Magnification 63 × ; scale bar 10 μm. [file Image_2.TIFF]

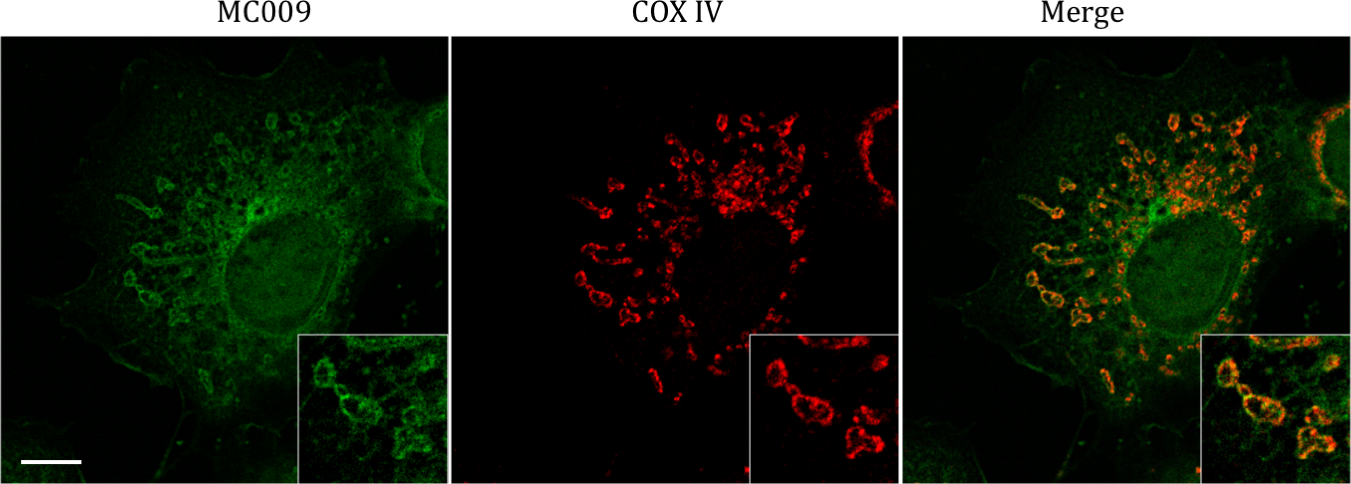

Supplement: Supplementary Figure 3 — Mitochondrial markers and MC009 co-localization in LX2 fixed cells. LX2 fixed cells immunostained for the mitochondrial membrane protein COX-IV; cells were counter-labeled with MC009. Scale bar 10 μm. [file Image_3.TIFF]

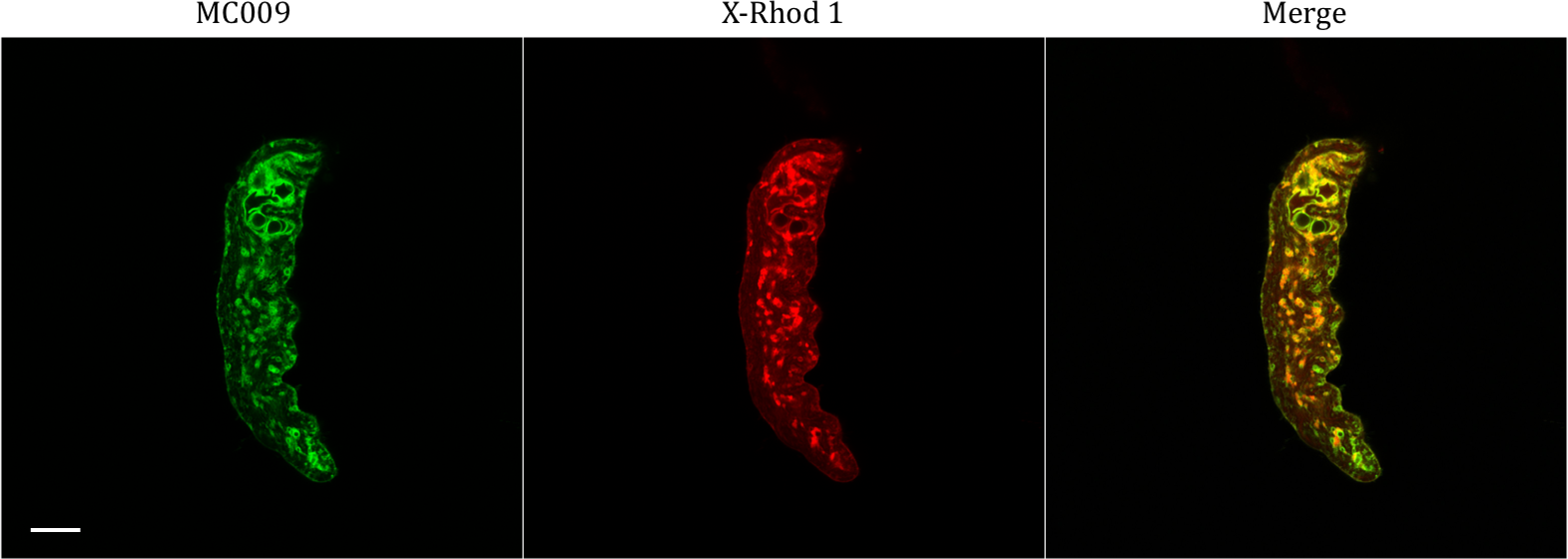

Supplement: Supplementary Figure 4 — Distribution of MC009 in mouse vascular smooth muscle cells. Live imaging of mouse primary vascular smooth muscle cells labeled with MC009 and counterstained with the calcium indicator X-Rhod-1™. Magnification 63 × ; scale bar 10 μm. [file Image_4.TIFF]
